# Small changes in synaptic gain lead to seizure-like activity in neuronal network at criticality

**DOI:** 10.1038/s41598-018-37646-9

**Published:** 2019-01-31

**Authors:** Jiaxin Du, Viktor Vegh, David C. Reutens

**Affiliations:** The University of Queensland, Centre for Advanced Imaging, St Lucia, QLD 4072 Australia

## Abstract

Epilepsy is a neurological disorder characterised by spontaneous recurrent seizures. The mechanisms by which multiple molecular and cellular changes lead to seizures is not well understood. Here, we study cortical seizure generation by simulating the activity of neuron groups in a network using the laminar cortex model. We identified a clear boundary between low-amplitude, asynchronous activity and high-amplitude, rhythmic activity, around which small changes in excitatory synaptic gain led to strong oscillatory activity. Neuron groups only responded significantly to stimulation around the boundary. The consequences of biophysical changes induced by epilepsy-related SCN1A mutations were also examined. Marked reduction in neuronal inhibition, as caused by mutations underlying Dravet syndrome, invariably led to strong neuronal firing, whereas small reductions in inhibition could cause significant changes when the network was poised close to the boundary. The study highlights the critical role of network dynamics in seizure genesis.

## Introduction

Recurrent spontaneous seizures, which are characterised by hyper-synchronous neuronal discharges^1,2^, are a defining feature of epilepsy. This disorder affects about 50 million people globally^3^, and in about 30%, seizures remain refractory to antiepileptic drugs. To develop new treatment strategies, it is vital to understand how seizures are initiated and propagate in the brain. Animal models in which seizures are induced using pharmacological, physical, or genetic approaches, have played a major role in advancing our understanding of the pathophysiology of seizures^4^. Studies in these models have linked multiple molecular and cellular mechanisms to seizure generation^5^. How these mechanisms modulate neuronal function has been studied extensively, but effects on the activity of the neuronal network as a whole, which requires simultaneous recording from a large number of neurons, are less well understood. Computational modelling is a powerful tool for understanding brain activity across scales, extending from the level of the ion channel to individual neurons and small and large-scale neuronal networks^6–8^. By modelling the activity of a large number of connected neurons under different physiological conditions and with different synaptic connections, computer simulations may reveal the network properties and mechanisms underlying the genesis of seizures and epilepsy^9^. Informative computational models need to consider the characteristics of neurons and synapses while simulating the collective activity of the neuronal network.

Mechanisms of seizure generation are likely to vary in different epilepsy phenotypes, reflecting the interplay of multiple contributors including pyramidal neurons, interneurons, and astrocytes and changes affecting synaptic connections, gap junctions, and extracellular ion concentrations^10^. Seizure generation can be thought of in terms of an episodic shift in the balance of inhibition and excitation. Several mutations in pre- and post-synaptic proteins are epileptogenic, supporting the idea that these shifts in balance arise from abnormal short-term synaptic plasticity over seconds, transforming high-normal levels of network activity into ictal activity^10,11^. Activity-dependent disinhibition may also result from dysregulation of intra- and extracellular concentrations of ions including potassium, calcium, protons, and chloride. For example, sustained stimulation of GABAergic synapses due to intense firing of interneurons is thought to lead to progressive accumulation of the chloride ion within postsynaptic neurons resulting in hyperpolarization followed by sustained depolarization caused by outward HCO_3_^−^ currents^12^. Intense neuronal firing also induces potassium efflux via the potassium-chloride cotransporter, KCC2, increasing potassium (K^+^) concentration in the extracellular space and contributing to increased neuronal excitation^13^.

In the present paper, we study cortical seizure generation using computer simulations based on the laminar cortex model (LCM), a computational framework capable of simulating the activity of neuron groups in cortical columns^14,15^. The LCM reflects many features of neuron groups and incorporates a realistic cortical architecture. It has been used to simulate the local field potentials (LFP) of the visual cortex under different conditions of visual stimulation^14^ and was able to produce spontaneous LFPs exhibiting frequency-inverse (1/f) power spectrum behaviour. Non-specific stimulation enhances gamma frequency oscillation in the model. The LCM also captures the fundamental as well as high order harmonics in LFPs during simulated intermittent light stimulation. We employed the LCM to examine the impact of changes in neuronal excitation and inhibition on the firing rates, oscillation state and synchronization of neuron groups. We also examined the effects of biophysical changes associated with mutations in the SCN1A gene that cause epilepsy.

## Results

### Architecture of the LCM

**Figure 1:**
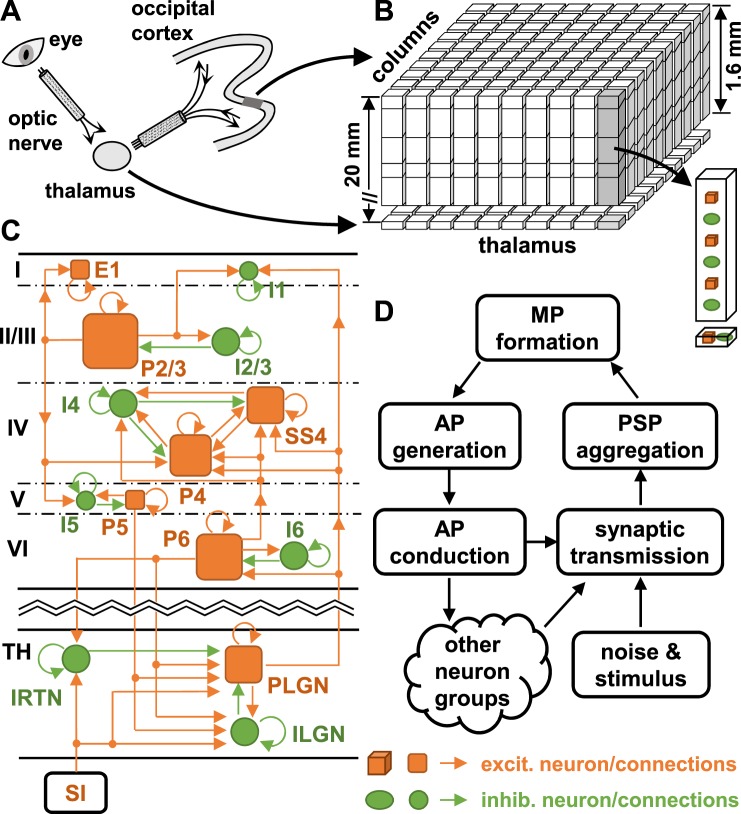
Architecture of the LCM. Illustrated are (**A**) the visual pathway in cats, (**B**) the structure of the LCM, (**C**) the synaptic connections between neurons simulated in the LCM, and (**D**) the neuronal processes simulated in the LCM. In Figure (**C**), the arrows indicate the direction of synaptic transmission; and sizes of squares and circles indicate the relative densities of corresponding neuron groups (not to scale). Only a partial synaptic connection map is shown; a complete map is provided in Table S5 in the Supplementary Information. Acronyms: **SI**–sensory input; **TH**–thalamus; **AP**–action potential; **PSP**–postsynaptic potential; **MP**–membrane potential; **E1**–excitatory neurons in layer I of the cortex; **I1**–interneurons in layer I; **P2/3**–pyramidal neurons in layer II and III; **I2/3**–interneurons in layer II and III; **P4**–pyramidal neurons in layer IV; **SS4**–spiny stellate neurons in layer IV; **I4**–interneurons in layer IV; **P5**–pyramidal neurons in layer V; **I5**–interneurons in layer V; **P6**–pyramidal neurons in layer VI, **I6**–interneurons in layer VI; **IRTN**–interneurons in RTN of the thalamus; **RLGN**–relay neurons in LGN of the thalamus; **ILGN**–interneurons in LGN.

For the reader’s convenience, we introduce the structure of the LCM (see Fig. 1). The LCM represents a small region in the visual cortex of the cat as a sheet of cortical columns, within which neuron groups are situated (see Methods and Fig. 1B). Neuronal dynamics are modelled using a ‘mean field approximation’ approach, which treats the same type of neurons within a column as a group acting as a single entity within the network. The dynamics and connections of single neurons in a neuron group are averaged using the mean field approximation^14,16^. We expanded the original LCM to incorporate a thalamocortical loop. Hence, the model now incorporates eleven neuron types in the cortex and three neuron types in the thalamus (see Fig. 1C,D and Table S1). Interactions between neuron groups are controlled by synaptic connections within the cortex and the thalamus (see Fig. 1C and Table S5). The LCM models the stimulus input to the thalamus as point spike sources, with spike rates generated using a Monte Carlo method (see Methods). For each run of the LCM, the membrane potentials of all neuron groups are first initialised at their resting values (−65 V), and then updated every 0.5 msec through five processes: action potential (AP) generation, AP conduction and distribution, synaptic transmission, postsynaptic potential (PSP) aggregation, and membrane potential (MP) formation (see Fig. 1D). Each of these processes is modelled using a set of equations elaborated upon in the Methods. The results reported below were generated by an instantiation of the LCM configured to simulate a cortical area of 1.12 × 1.12 mm^2^, which contains 20 × 20 cortical columns with the size of 56 μm^17^. The results were produced from a 5.12-second recording of neuronal firing rates after 10 seconds of running of the model.

### Temporal features of different states of neuronal activity

Parameters used to model the behaviour of neuron groups were derived from published experimental results (See Tables S1–S5). We found that the excitatory and inhibitory synaptic gains (*g*_E_ and *g*_I_), which respectively control the efficiency of excitatory and inhibitory synaptic transmission, dramatically influence neuronal firing rates. Mathematically, synaptic gain can be expressed as (also see Equation (7)),

1 $${g}_{p}=\frac{PS{P}_{qp}}{{\varphi }_{p}{N}_{qp}}f({V}_{q},{\varphi }_{p})$$

where *PSP*_*qp*_ is the amplitude of PSPs at an afferent spike rate *ϕ*_*p*_, *N*_*qp*_ is the number of synapses, and *f*(*V*_*q*_, *ϕ*_*p*_) is a scaling factor for spike adaptation and membrane potential dependency (refer to Equation (7) for more details). From a network perspective, excitatory and inhibitory synaptic gain respectively reflect the excitatory and inhibitory connection strength between neurons in the network with changes in gain corresponding to the effects of short-term synaptic plasticity.

**Figure 2:**
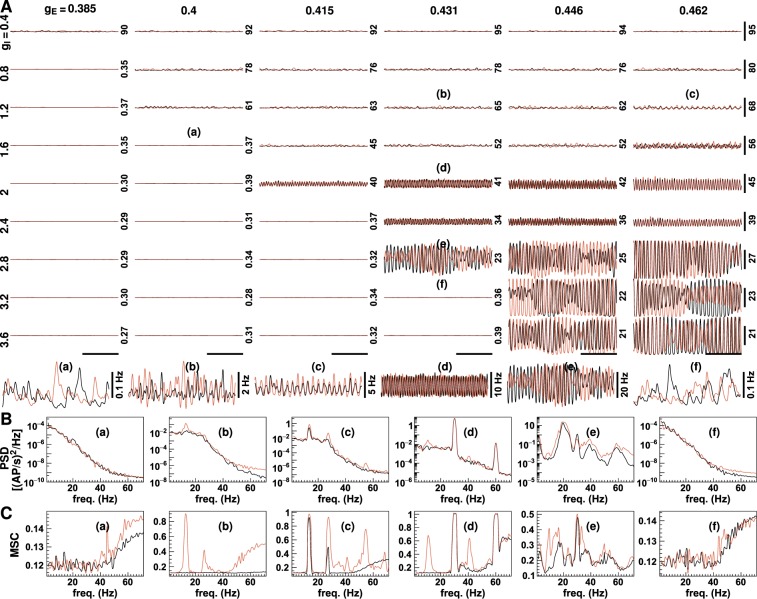
Neuronal firing rates with different synaptic gains. The plots in panel (A) displays firing rates simulated without (black lines) and with stimulus (red lines) using a range of excitatory and inhibitory synaptic gains. The excitatory and inhibitory synaptic gain values are shown on the top and the left, respectively [unit: uV/(mV · Hz)]. The number on the right of each figure indicates the value at the middle of the vertical scale bars (unit: AP/sec). The vertical scale bars on the right represent a firing rate of 20 AP/sec, and the horizontal scale bars at the bottom represent a period of 500 msec. Six amplified firing rate traces are plotted below the panel. The figures in panels (B and C) show the power spectrum densities (PSD) and magnitude-squared coherence (MSC) of six typical neuronal firing rates in panel (A). Corresponding plots are indicated by the lower case letters (a–c etc.). See also Fig. 3.

Figure 2 displays the neuronal firing rates for a range of synaptic gains. Three types of neuronal activity with markedly different firing rates and oscillation states were noted. Low amplitude asynchronous activity occurred at low excitatory synaptic gain [<0.38 uV/(mV · Hz)] or high inhibitory synaptic gain (see Fig. 2). Here, the mean firing rate was less than one AP/sec. Neuronal activity was of low frequency with no specific frequency being dominant. High amplitude asynchronous activity was produced when the excitatory synaptic gain was high and the inhibitory gain was small. The mean firing rate exceeded 60 AP/sec, and in the frequency spectrum of neuronal firing rates, low-frequency oscillations were enhanced. When both excitatory and inhibitory synaptic gain were large, strong rhythmic activity occurred. In this state, the neurons were highly activated (mean firing rates >10 AP/sec), and the frequency spectra showed obvious peaks.

**Figure 3:**
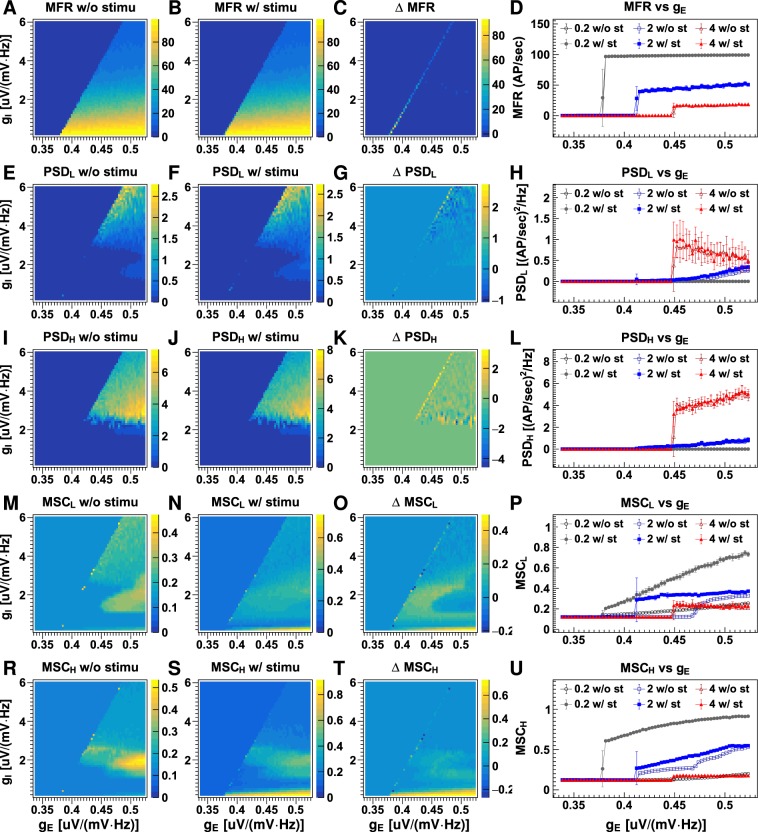
Neuronal firing rate properties with different synaptic gains. The plots display the mean firing rates (MFR) without stimulation (**A**), with stimulation (**B**), their differences (**C**) and dependency on excitatory gain for three typical inhibitory gains (**D**); the mean power spectrum density over firing rates of 2–15 Hz (PSD_L_) without stimulation (**E**), with stimulation (**F**), their differences (**G**) and dependency on excitatory gain (**H**); the mean power spectrum density over firing rates of 16–50 Hz (PSD_H_) without stimulation (**I**), with stimulation (**J**), their differences (**K**) and dependency on excitatory gain (**L**); and the mean magnitude-squared coherence for firing rates of 2–15 Hz (MSC_L_) without stimulation (**M**), with stimulation (**N**), their differences (**O**) and dependency on excitatory gain (**P**); the mean magnitude-squared coherence for firing rates of 16–50 Hz (MSC_H_) without stimulation (**Q**), with stimulation (**R**), their differences (**S**) and dependency on excitatory gain (**T**). Discrete values for synaptic gain were used with minimum intervals of 0.003 uV/(mV · Hz) and 0.1 uV/(mV · Hz) between adjacent values for excitatory and inhibitory synaptic gain respectively. On the right column, the lines with open and closed markers display results for datasets without and with stimulus, respectively. Error bars on the line plots show the standard deviations (SD) for 10 runs of the LCM with the same parameter values but different random number kernels. See also Fig. 4.

**Figure 4:**
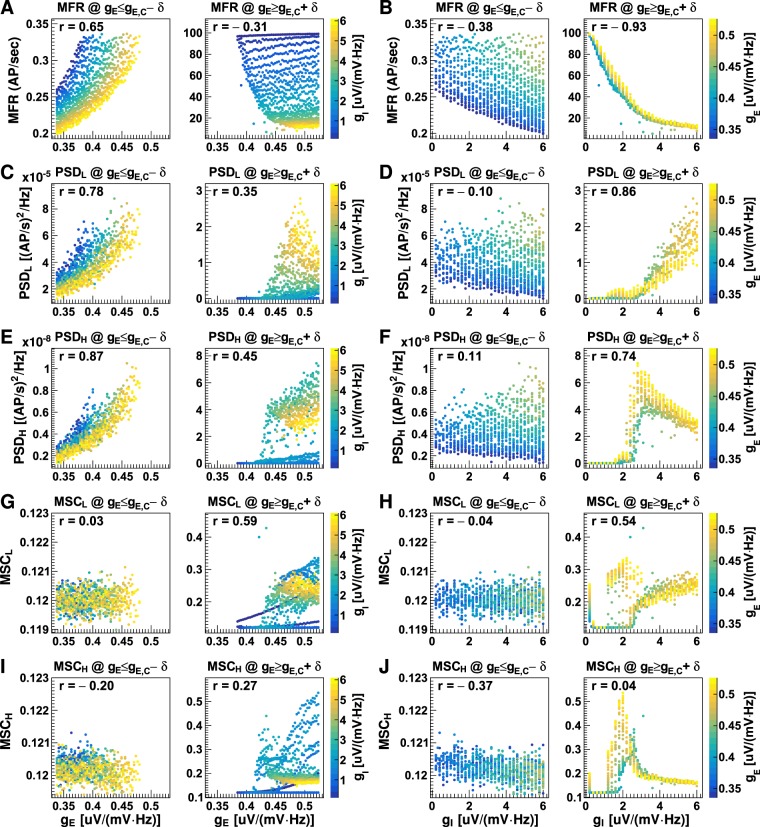
Neuronal firing rate measurements plotted versus synaptic gains. Shown are the mean firing rates (MFR; (**A**,**B**)), the mean power spectrum density over 2–15 Hz (PSD_L_; (**C** and **D**)), the mean power spectrum density over 16–50 Hz (PSD_H_; (**E** and **F**)), the mean magnitude-squared coherence over 2–15 Hz (MSC_L_; (**G**,**H**)), the mean magnitude-squared coherence over 16–50 Hz (MSC_H_; (**I** and **J**)) of neuronal firing rates plotted versus excitatory gain (**A**,**C,E,G** and **I**) and inhibitory gain (**B**, **D,F,H** and **J**) for conditions when excitatory gains were smaller than the critical values (*g*_E_ ≤ *g*_E,C_ − *δ*, where *δ* = 0.002 uV/(mV · Hz)) and when excitatory gains were larger than the critical values (*g*_E_ ≥ *g*_E,C_ + *δ*). Each dot on the plot represents a result obtained using one synaptic gain pair. The inhibitory synaptic gain values in figure (**A**,**C,E,G** and **I**) and the excitatory synaptic gain values in figure (**B**,**D,F,H** and **J**) are represented by colour, and the corresponding colour bars are shown on the right. The Pearson’s correlation coefficient (r) was calculated for each case. The data was produced from non-stimulated datasets, as shown in Fig. 3.

Figure 3 displays four quantities calculated from the neuronal firing rates obtained using different synaptic gains: temporal mean firing rates (MFR), the mean power spectrum densities (PSD) of the low (2–15 Hz, PSD_L_) and the high (16–50 Hz, PSD_H_) frequency bands, the mean magnitude-squared coherence (MSC) of the low (MSC_L_) and the high (MSC_H_) frequency band. The first three measurements quantify temporal properties of neuronal firing and the MSCs measure coherence between neuron firing in columns. In Fig. 4, parameters are plotted against excitatory and inhibitory synaptic gains. Figures 3 and 4 reveal a complex relationship between neuronal firing rate and synaptic gains. The firing rate generally increases as excitatory synaptic gain (*g*_E_) increases or inhibitory synaptic gain (*g*_I_) decreases in magnitude. However, the change in firing rate is not continuous; a series of critical synaptic gain combinations are observed, around which small changes in excitatory or inhibitory gain cause dramatic jumps in the firing rate. For example, with an inhibitory gain of 1 uV/(mV · Hz), the firing rate jumps from about 0.3 AP/sec to >60 AP/sec as excitatory gain increases from 0.394 to 0.397 uV/(mV · Hz) (refer to Fig. 3D). On the synaptic gain maps shown in Fig. 3, the critical combinations of excitatory and inhibitory synaptic gains fall along a line:

2 $${g}_{{\rm{E}},{\rm{C}}}=0.018{g}_{{\rm{I}}}+0.376,\,{\rm{or}}\,{g}_{{\rm{I}},{\rm{C}}}=55.6{g}_{{\rm{E}}}+20.89,$$

where *g*_E,C_ and *g*_I,C_ are the critical excitatory and inhibitory gains, respectively. Secondly, this line divides neuronal firing rates into two regions: Region 1: where *g*_E_ < *g*_E,C_ and *g*_I_ > *g*_I,C_, in which the neurons fire at low rate (mean firing rate <0.4 AP/sec), and Region 2: where *g*_E_ > *g*_E,C_ and *g*_I_ < *g*_I,C_, in which the neurons fire at medium to high rates (mean firing rate >10 AP/sec, refer to Fig. 4A,B). Neuronal firing rates in the two regions exhibit different dependencies on synaptic gains. In Region 1, the firing rate is positively correlated with both the excitatory (Pearson’s correlation coefficient r = 0.65) and the inhibitory gain (r = 0.38). In Region 2, firing rate is weakly negatively correlated with excitatory gain (r = −0.31), but strongly negatively correlated with inhibitory gain (r = −0.93). Neuronal firing rate increases exponentially as the magnitude of inhibitory gain decreases. The firing rate increases from about 20 AP/sec at *g*_I_ = 6 uV/(mV · Hz) to 40 AP/sec at *g*_I_ = 2 uV/(mV · Hz) and about 100 AP/sec at *g*_I_ = 0.2 uV/(mV · Hz). Excitatory gain does not affect the firing rate significantly (see Fig. 4B), but the range of firing rates increases slightly with a decrease in the lower limit (refer to Fig. 4A).

In Region 1 (*g*_E_ < *g*_E,C_), oscillations in both low and high frequency bands increased with excitatory gain (r = 0.78 for low frequency band; and r = 0.87 for high frequency band; refer to Fig. 4C and E), and were not significantly affected by changes in inhibitory gain (r = −0.1 for low frequency band; and r = 0.11 for high frequency band; refer to Fig. 4D and F). In Region 2 (*g*_E_ > *g*_E,C_), the PSDs remained low [<0.1 (AP/sec)^2^/Hz] for excitatory gain <0.43 uV/(mV · Hz). When excitatory gain exceeded 0.43 uV/(mV · Hz), the PSDs varied between 0–2 (AP/sec)^2^/Hz for the low-frequency band and 0–8 (AP/sec)^2^/Hz for the high-frequency band. As with neuronal firing rate, the PSDs were more strongly dependent on inhibitory gain than on excitatory gain (refer to Fig. 4C–F). PSDs in both low and high-frequency bands increased as inhibitory gain increased, implying that strong neuronal oscillations require high inhibitory activity. The PSDs of high but not low-frequency bands appeared to saturate at *g*_I_ = 3 uV/(mV · Hz) (refer to Fig. 4F).

The MSCs in both low and high-frequency bands did not vary significantly with either excitatory or inhibitory gains in Region 1 (*g*_E_ < *g*_E,C_). In Region 2 (*g*_E_ > *g*_E,C_), the correlations between the MSCs and excitatory gains were moderate in the low-frequency band (r = 0.59) and low in the high-frequency band (r = 0.27). The MSCs had a strong, non-linear relationship with the inhibitory gains in the region. Highly coherent neuronal firing occurred at high excitatory gain [*g*_E_ ≥ 0.43 uV/(mV · Hz)] and moderately low inhibitory gain (between 1.3 and 3.3 uV/(mV · Hz); refer to Fig. 4G,H).

The LCM incorporates spike inputs from two external sources: cortico-cortical projections to cortical neurons and sensory inputs to thalamic neurons. Cortico-cortical input was modelled as low-magnitude (1 AP/sec), low-frequency (frequency cut off at 20 Hz) white noise resembling temporal features of alpha activity generated in the absence of a stimulus. Afferent thalamic input was modelled for two conditions: (1) the non-stimulated state, modelled as low magnitude (1 AP/sec) low frequency (frequency cut off at 20 Hz) white noise; and (2) the stimulated state, modelled as high magnitude (50 AP/sec) high frequency (frequency cut off at 50 Hz) white noise. Cortico-cortical connections to each column were treated as being independent (i.e., asynchronous input) whereas thalamic inputs were the same for all columns (i.e., synchronous input). The neuronal firing rates and their response to stimulation are shown in Figs 2 and 3. Stimulation did not change mean firing rates significantly when ${g}_{{\rm{E}}}\gg {g}_{{\rm{E}},{\rm{C}}}$ or ${g}_{{\rm{E}}}\ll {g}_{{\rm{E}},{\rm{C}}}$ but firing rate increased by as much as 80 AP/sec when *g*_E_ was close to *g*_E,C_ (see Fig. 3C). Stimulation did not affect neuronal oscillation or synchronisation in Region 1 (*g*_E_ < *g*_E,C_). In Region 2 (*g*_E_ > *g*_E,C_), stimulation changed PSDs of neuronal firing in both low and high-frequency bands only when *g*_I_ > 2.4 uV/(mV · Hz) (Fig. 3G and K) and it significantly increased MSCs of neuronal firing in both low and high-frequency bands when *g*_*I*_ < 0.4 uV/(mV · Hz) (refer to Fig. 3O and S).

### Response to transient changes in synaptic gain

**Figure 5:**
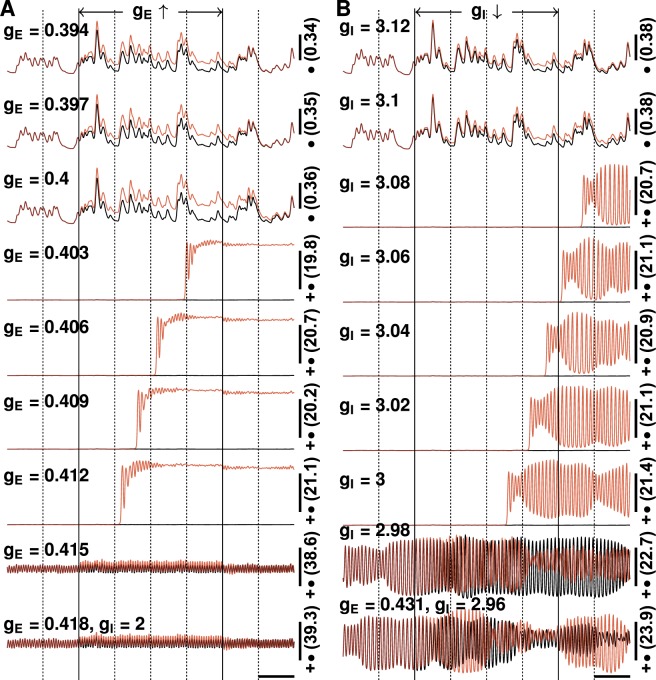
Neuronal firing rates under a small transient change in synaptic gains. (**A**) The red lines display the firing rates when the excitatory synaptic gains, which are shown on the left, are increased by 0.015 uV/(mV · Hz) for 2 seconds (indicated by solid vertical lines). The black lines display neuronal firing rates without the change in synaptic gain. Inhibitory gains are fixed at 2 uV/(mV · Hz) and the corresponding critical excitatory gain is ~0.431 uV/(mV · Hz). (**B**) The red lines display the firing rates when inhibitory synaptic gains, which are shown on the left, are reduced by 0.2 uV/(mV · Hz) (i.e., weaker inhibition) for 2 seconds (indicated by solid vertical lines). The black lines display neuronal firing rates without the change in synaptic gain. The excitatory gains are fixed at 0.431 uV/(mV · Hz) and the corresponding critical inhibitory gain is about 3 uV/(mV · Hz). The number on the right is the firing rate at the middle of the vertical scale bar (unit: AP/sec), and markers represent the values of vertical scale bars: •**−**0.1 AP/sec, ••**−**0.5 AP/sec, **+−**5 AP/sec; **+**•**−**25 AP/sec, **++−**50 AP/sec. For comparison, the same random number kernels were used for all cases.

The results shown above were simulated using fixed synaptic gains. However, the efficiency of synaptic transmission may be influenced by many factors. We therefore examined the changes in neuronal firing rates induced by slight, transient changes in synaptic gain (see Fig. 5). For most combinations of synaptic gains, a change in firing rate occurred within milliseconds and neuronal activity returned to its previous state as soon as gains were restored to their initial values. The transition was more complex, however, for certain combinations of synaptic gain. For example, for the combination of g_E_ = 0.4 uV/(mV · Hz) and g_I_ = 2 uV/(mV · Hz) (see Fig. 5), the effects of changes in excitatory gain persisted for hundreds of milliseconds. For the combination of g_E_ = 0.403 uV/(mV · Hz) and g_I_ = 2 uV/(mV · Hz), a 0.015 uV/(mV · Hz) increase in excitatory gain resulted in significant changes in neuronal activity only after a 1-second delay with a strong neuronal oscillation that lasted almost 0.5 second before steady state was reached. Neuronal activity did not return to its original state after synaptic gains were restored to their initial values. Similar behaviours were also observed with temporary changes in inhibitory synaptic gain. It should be noted that the complex transitions in neuronal activity occurred when *g*_E_ increased from below *g*_E,C_ to above *g*_E,C_ or *g*_I_ decreased from above *g*_I,C_ to below *g*_I,C_.

### Response to changes in the reversal potential of inhibitory synapses

**Figure 6:**
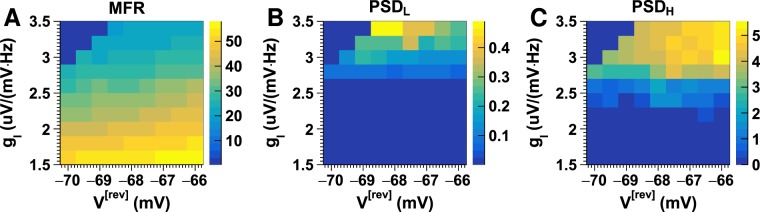
Neuronal firing rate measurements under a small shift in the reversal potentials of inhibitory synapses. Shown are the mean firing rates (MFR; (**A**)), the mean power spectrum density over 2–15 Hz (PSD_L_; (**B**)), and the mean power spectrum density over 16–50 Hz (PSD_H_; (**C**)) simulated using combinations of inhibitory synaptic gains (*g*_*I*_) and reversal potentials of inhibitory synapses (${{V}_{I}}^{[{\rm{rev}}]}$).

We also examined neuronal network response to changes in the reversal potentials of inhibitory synapses. Figure 6 displays neuronal firing rates obtained using different combinations of inhibitory synaptic gains and reversal potentials of inhibitory synapses. We observed a boundary in the map, around which a small positive shift in the reversal potential led to a jump in neuronal firing rates (Fig. 6A). The critical values of the reversal potentials moved positively when inhibitory gain increased. In addition, neuronal oscillations did not change significantly with the reversal potential under weak inhibition but the oscillation amplitudes in both low- and high-frequency bands increased significantly when reversal potentials shifted positively (Fig. 6B,C).

### Neuronal activity with changes associated with SCN1A mutations

The LCM can be used to analyse the behaviour of the network with changes in the biophysical properties or synaptic connections of neurons. We examined the effects of biophysical changes associated with SCN1A gene mutations. This gene encodes the alpha 1 subunit (Nav1.1) of the neuronal voltage-gated sodium channel. Mutations are associated with a wide range of epilepsy syndromes, ranging from relatively mild simple febrile seizure (FS) and Generalised Epilepsy with Febrile Seizures Plus (GEFS+) to more severe Intractable Childhood Epilepsy with Generalized Tonic-Clonic seizures (ICEGTC) and Dravet syndrome (DS)^18–22^. A variety of biophysical changes have been associated with different mutations, including impaired firing capability and positive shifts in the firing thresholds of inhibitory neurons^23–25^.

**Figure 7:**
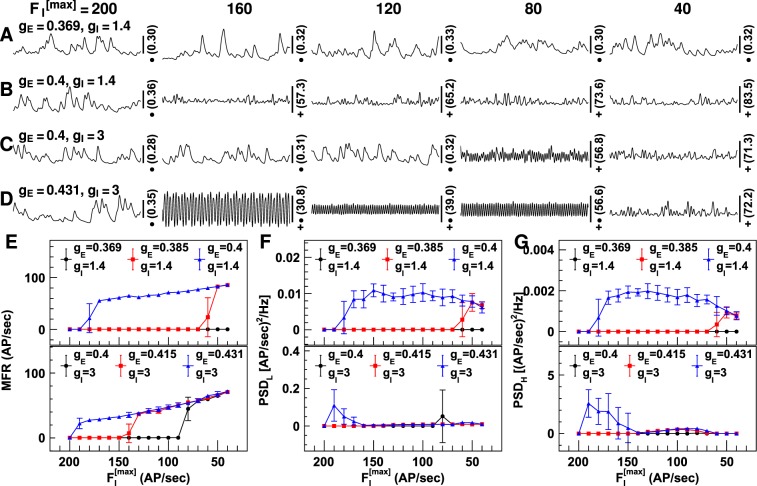
Neuronal firing rates with reduced firing capability of inhibitory neuronal groups. Shown are the neuronal firing rates when the maximum firing rate of inhibitory neuron groups (${{F}_{{\rm{I}}}}^{[{\rm{\max }}]}$) is reduced. Figures (**A**–**D**) show the firing rates simulated using four combinations of synaptic gains. The synaptic gain values are shown on the left [unit: uV/(mV · Hz)] and ${{F}_{{\rm{I}}}}^{[{\rm{\max }}]}$ values are shown on the top of the figures (unit: AP/sec). The number on the right is the firing rate at the middle of the vertical scale bar (unit: AP/sec), and the markers indicate the values of vertical scale bars: •**−**0.1 AP/sec; **+−**5 AP/sec; **+**•**−**25 AP/sec. The horizontal scale bars represent a 200 millisecond period. Figures (**E**–**G**) display the temporal mean firing rates (MFR; (**E**)), and the mean power spectrum density for firing rates of 2–15 Hz (PSD_L_; (**F**)) and 16–50 Hz (PSD_H_; (**G**)). The synaptic gain values are displayed on the top [unit: uV/(mV · Hz)]. The error bars represent the standard deviation for 10 runs of the LCM with the same parameter values but different random number kernels.

Impaired firing capability was simulated by gradually reducing maximum firing rates ${{F}_{{\rm{I}}}}^{[{\rm{\max }}]}$ (see Equation (5)) from 200 to 40 AP/sec, in steps of 10 AP/sec, in all inhibitory neuron groups. Figure 7 displays the neuronal firing rates with reduced ${{F}_{{\rm{I}}}}^{[{\rm{\max }}]}$ and different synaptic gains. As expected, the neuronal firing rate increased as ${{F}_{{\rm{I}}}}^{[{\rm{\max }}]}$ decreased for all synaptic gains tested, but the firing rate did not change continuously. It jumped from a low rate to a high rate around certain values of ${{F}_{{\rm{I}}}}^{[{\rm{\max }}]}$, and the closer *g*_E_ was to *g*_E,C_, the smaller the reduction in ${{F}_{{\rm{I}}}}^{[{\rm{\max }}]}$ required for an abrupt transition (refer to Fig. 7E). PSDs at both high and low frequency increased as ${{F}_{{\rm{I}}}}^{[{\rm{\max }}]}$ was reduced and there was an abrupt transition at the same ${{F}_{{\rm{I}}}}^{[{\rm{\max }}]}$ as that at which the abrupt transition in firing rates was observed. PSDs at both high and low frequency decreased when ${{F}_{{\rm{I}}}}^{[{\rm{\max }}]}$ was extremely low (${{F}_{{\rm{I}}}}^{[{\rm{\max }}]}$ < 70 AP/sec).

**Figure 8:**
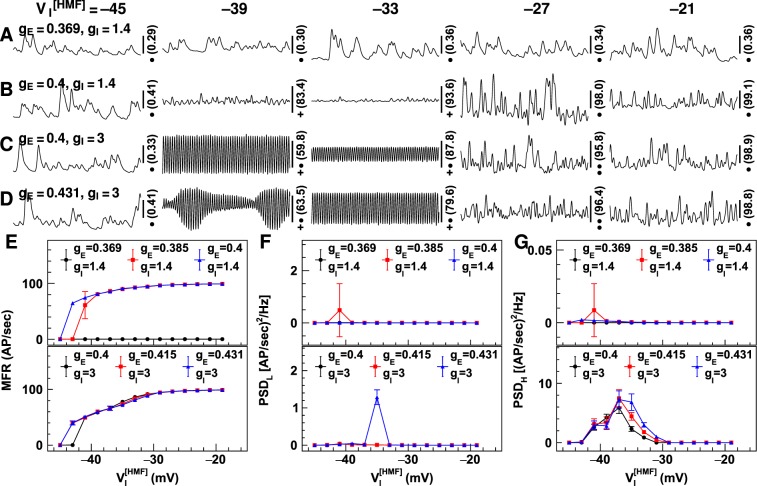
Neuronal firing rates simulated using positively shifted firing thresholds of inhibitory neuron groups. Shown are the neuronal firing rates simulated using positively shifted voltage at half-maximum firing (${V}_{{\rm{I}}}^{[{\rm{HMF}}]}$). Figures (**A**–**D**) display the firing rates obtained using four synaptic gain combinations. The synaptic gain values are shown on the left [unit: uV/(mV · Hz)], and the values for ${V}_{{\rm{I}}}^{[{\rm{HMF}}]}$ are shown on the top (unit: mV). The number on the right is the firing rate in the middle of the vertical scale bar (unit: AP/sec), and the markers indicate the values of vertical scale bars: •**−**0.1 AP/sec; ••**−**0.5 AP/sec; **+−**5 AP/sec; **+**•**−**25 AP/sec. The horizontal scale bars represent a 200 millisecond period. Figures (**E**–**G**) display the temporal mean firing rates (MFR; (**E**)), and power spectrum density for firing rates of 2–15 Hz (PSD_L_; (**F**)) and 16–50 Hz (PSD_H_; (**G**)) using different synaptic gains. Synaptic gain values are displayed at the top [unit: uV/(mV · Hz)]. Error bars represent the standard deviation for 10 runs of the LCM using the same parameter values but different random number kernels.

A positive shift in the firing thresholds of inhibitory neurons was simulated by gradually increasing the voltage at half-maximum-firing (${{V}_{{\rm{I}}}}^{[{\rm{HMF}}]}$; see Equation (5)) for all inhibitory neuron groups from −45 mV to −19 mV in steps of 2 mV. Figure 8 displays the neuronal firing rates with changes in ${{V}_{{\rm{I}}}}^{[{\rm{HMF}}]}$ and different synaptic gain combinations. Positive shifts in firing thresholds caused the neuronal firing rate to increase discontinuously. Even a small, 2–4 mV, shift in neuronal firing threshold caused a significant increase in the firing threshold (refer to Fig. 8E). The PSDs at both high and low frequencies increased after a small positive shift in firing threshold, but they returned to minimum values for ${{V}_{{\rm{I}}}}^{[{\rm{HMF}}]}$ > −29 mV.

## Discussion

In this paper, we study the neuronal activity in the cortex using computer simulations based on the LCM. The LCM incorporates many realistic features of neuronal networks allowing us to gain insights into neuronal dynamics under various conditions that are relevant to the generation of seizures.

### Neuronal network dynamics

For the normal function of a brain network, outputs (neuronal firing rates) associated with different input conditions (stimuli) should be distinguishable from each other. Our results suggest that this is the case when synaptic gains are close to their critical values (i.e., *g*_E_ close to *g*_E,C_). In the cortex, thalamic projections account for less than 5% of the total synapses on a neuron (refer to Table S5 in the Supplementary Information) and are greatly outnumbered by distant cortico-cortical projections and local connections. Spike inputs from the thalamus are unlikely to evoke significant responses in the firing states of the cortical neurons unless their membrane potentials are already poised close to the firing threshold. Therefore, the neuronal network must maintain some level of activity even in the absence of the stimulus. This may help to explain previous observations that noise may help to improve the function of neuronal networks (see, for example, paper of Hansel and Vreeswijk^26^).

The neuronal network exhibited significantly different dynamics in the two synaptic gain regions, indicating that transitions of neuronal network phases occurred around the boundary between the two regions. The network in Region 1 (*g*_E_ < *g*_E,C_) is in a disordered phase, because the activity of neuron groups is more strongly influenced by noise than by neighbouring neurons. The network in Region 2 (*g*_E_ > *g*_E,C_) operates in an ordered phase because the activity of neuron groups is highly correlated. Our results suggest that the neuronal network of the brain operates in a balanced critical state between the two phases. This finding aligns with the proposal that neuronal networks in the brain operate in a self-organised critical state^27,28^. The criticality hypothesis has gained support from both theoretical considerations and experimental observations. Theoretically, a neuronal network in a critical state can maintain some order to ensure consistent responses to specific stimuli but allows a degree of disorder to enable adaptation to variations in external conditions^29^. Experimentally, neuronal avalanches with an inverse power-law distribution for avalanche size and duration, a characteristic of systems in a self-organised critical state, have been observed in the spontaneous activity of organotypic neuronal cultures from rats^28,30^ and humans^31^. In diseased tissue, such as that from patients with epilepsy, neuronal avalanches deviate from the power-law distribution^32^. Our simulations provide further compelling support for the criticality hypothesis insofar as neuronal networks only show a significant response to stimuli when they are situated close to the critical state (refer to Fig. 3).

Many interesting behaviours were observed when the network was close to the critical state. Firstly, the neuronal network in a critical state displayed some degree of adaptation to changes in neuronal excitation and inhibition (refer to Fig. 4). The network first reacted to increased neuronal excitation with strong oscillatory activity. Then, it gradually absorbed the changes by increasing firing, and oscillation amplitude fell significantly. The observed adaptation may help to clarify a long-standing conundrum: how does a neuronal network at criticality avoid slipping into either a completely disordered or a completely ordered state in a noisy environment. Our results suggest that the neuronal network itself is capable of absorbing temporal changes in neuronal excitation and inhibition. This, together with adaptation in synaptic transmission and the refractory period of neuron firing, may confer tolerance in the network to significant fluctuations in background activity. Secondly, the network showed hysteresis – network responses were delayed (by about one second) after the change in synaptic gain (refer to Fig. 4). The hysteresis phenomenon is unlikely to be caused by AP propagation along axons or conduction of PSPs along dendrites, because the former only lasts several milliseconds and the latter less than 100 milliseconds. A more likely cause for the delay is the cascade of neuronal excitability within the network (see below). This suggests that the network may be able to accumulate neuronal excitation for a period much longer than the duration of an AP or PSP, which may have important implications for the generation of seizures.

### Implications for seizure generation

Seizure initiation in the brain is a temporal dynamical process resulting from changes in neurons, synapses, and ion concentrations. Simulation of different states of the neuronal network predicts the changes in activity that are associated with changes in state. This can help to inform our understanding of how cellular and molecular changes can evoke or inhibit seizure generation.

Although it is possible for the neuronal network to achieve a critical state over a range of inhibitory activity, seizure vulnerability is greatest at a low level of inhibition. At a small inhibitory gain (<1 uV/(mV · Hz)), the rate of neuronal firing is close to maximal when the excitatory gain exceeds the critical value (see Fig. 3). The high firing rates persist even after excitatory gain falls below the critical value (further discussed below). Furthermore, stimulation significantly increased synchronisation when inhibitory gain was small. Seizure generation in neuronal networks with weak inhibition may result from the amplification of synchronised inputs in noisy environments.

**Figure 9:**
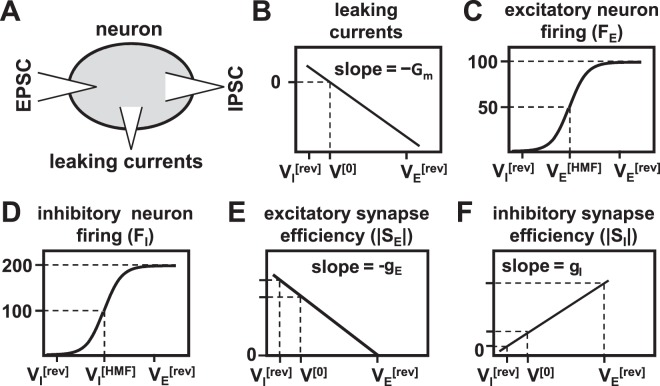
Factors that determine neuronal membrane potential. Illustrated are (**A**) a schematic diagram of currents flowing into and out of neurons, and the dependency of (**B**) leaking currents, (**C**) excitatory neuron firing rates (*F*_E_), (**E**) excitatory synapse efficiency (|*S*_E_|), (**D**) inhibitory neuron firing rates (*F*_I_), and (**F**) inhibitory synapse efficiency (|*S*_I_|) on membrane potentials. Synaptic efficiency is defined as $|{S}_{{\rm{p}}}|=|{g}_{p}({V}_{p}^{[\mathrm{rev}]}-{V}_{q})|$, where *g*_*p*_ is the synaptic gain, *V*_*q*_ is the membrane potential of the postsynaptic neuron, ${V}_{p}^{[\text{rev}]}$ is the reversal membrane potential and set to 0 mV for excitatory synapses and −70 mV for inhibitory synapses. The figures are plotted in arbitrary units. Acronyms: **EPSC**–excitatory postsynaptic currents, **IPSC**–inhibitory postsynaptic currents.

The simulations revealed distinctive roles of neuronal excitation and inhibition in regulating network dynamics. Excitation imposed a threshold on neuron firing with firing rates being lowest when neuronal excitation was below threshold and high firing rates when excitation exceeded the threshold. Above the threshold, inhibition plays an important role in controlling neuronal firing. The results can be explained by the interactions between three factors that determine neuronal membrane potential: leaking currents through the membrane, depolarisation caused by excitatory PSPs, and repolarisation caused by inhibitory PSPs (see Fig. 9A). Leaking currents reduce the membrane potential according to $u\cdot \exp (\,-\,t/{\tau }_{m})$, where *u* is the membrane potential above the resting potential, and *τ*_*m*_ is the membrane time constant (see, Equation (3) in Methods)^6^. The amplitudes of PSPs are influenced by neuron membrane potentials through two mechanisms. Firstly, the efficiency of excitatory synapses decreases and that of inhibitory synaptic transmission increases with the membrane potential of postsynaptic neurons. The relationships were modelled as linear functions in the LCM (see Equation (7) and Fig. 9E,F). Secondly, the afferent spike rates to synapses increase with the membrane potentials of presynaptic neurons (see Fig. 9C,D). The membrane potentials of pre- and postsynaptic neurons are highly correlated in the network due to the strong recurrent synaptic connections between neurons. The three factors play different roles in regulating neuron firing at different levels of excitatory gain. When the excitatory gain is low, PSP amplitudes do not change significantly because the afferent spike rates to synapses are low. However, leaking currents, which increase linearly with the membrane potential (see Fig. 9B), strongly restrain the growth of the membrane potential. As the excitatory gain increases, the amplitude of excitatory PSPs may outweigh the repolarisation caused by leaking currents, and membrane potentials of neurons begin to grow. When they exceed the firing thresholds, neuronal firing rates increase explosively, which leads to further depolarisation. The cascade of neuronal excitability occurs as long as the excitatory PSP amplitudes are higher than the membrane potential reduction due to leaking currents, and even a small net neuronal depolarisation eventually leads to explosive neuron firing. The cascade stops when the amplitudes of inhibitory PSPs match that of excitatory PSPs because increases in membrane potential reduce the efficiency of excitatory synapses and boost the efficiency of inhibitory synapses. The larger the inhibitory gain, the sooner the cascade stops. Therefore, the final membrane potentials depend more strongly on the inhibitory gain than on the excitatory gain.

Epilepsies associated with SCN1A mutations exhibit extraordinary clinical heterogeneity. Seizure manifestations vary between members of the same pedigree bearing identical mutations^33,34^. Previous studies have ascribed the phenotypic heterogeneity to the diversity of functional changes in the voltage-gated sodium channel caused by individual mutations^35,36^. Our simulations, however, suggest the effects of SCN1A mutations on the neuronal network depend on the levels of neuronal excitation and inhibition. Though severe loss of neuronal inhibition, as caused by the SCN1A mutation associated with DS, leads inevitably to high neuron firing, a small reduction in neuronal inhibition, as caused by the SCN1A mutation associated with the GEFS+ syndrome, only causes significant changes in neuronal activity when the network is poised close to the boundary. The mutation also causes other neuronal changes, such as changes in GABA_A_ chloride reversal potential, which may contribute to seizure generation^37^.

## Methods

In this section, we outline the structure of the LCM model, which enables cortical neuronal dynamics to be modelled under various conditions using model parameters that represent different neuronal behaviours. Additional details about the simulation methods and parameter values used in the paper are provided in the Supplementary Information.

### Laminar cortex model

The LCM was developed to simulate local field potentials (LFP) in the visual cortex of the cat. The LCM simulates neuronal activity of a flat sheet of cortical columns^14,15^. A cortical column may contain thousands of neurons, however, neurons in a column display similar responses to specific stimuli. Therefore, the LCM treats the same type of neurons within a column as a group which acts as a single entity in a network. A neuron group has similar features to a single neuron but its dynamics and connections are averaged using the mean-field approximation^14,16^. The approach allows us to model the neuronal activity of a large cortical region without knowing the detailed physiology of individual neurons.

In the current LCM, the evolution of neuronal membrane potentials is modelled using the following differential equation^38^

3 $${C}_{{\rm{m}}}\frac{du}{dt}=-\,{G}_{{\rm{m}}}u+{I}_{{\rm{E}}}$$

where *u* = *V* − *V*^[0]^ is the membrane potential relative to the resting state, and *V* and *V*^[0]^ are the membrane potential and resting membrane potential of neuron groups, respectively; *C*_m_ is the total capacitance of the membrane; *G*_*m*_ is total conductance of the membrane; the term −*G*_*m*_*u* represents the currents leaking through the membrane; and *I*_E_ is the total current flowing into the neuron through synaptic transmission, i.e., postsynaptic currents (PSC). The LCM calculates membrane potentials at discrete time points. For a small time interval, a simple iteration equation can be derived from Equation (3):

4 $$u(t+{\rm{\Delta }}t)\approx u(t){e}^{-{\rm{\Delta }}t/{\tau }_{m}}+{C}_{m}^{-1}{I}_{{\rm{E}}}{\rm{\Delta }}t=u(t){e}^{-{\rm{\Delta }}t/{\tau }_{m}}+{\rm{\Delta }}V(t),$$

where Δ*t* is the time interval; *τ*_*m*_ = *C*_*m*_/*G*_*m*_ is the membrane time constant characterising the time for the membrane to repolarize after depolarisation^6^; ${\rm{\Delta }}V={C}_{m}^{-1}{I}_{{\rm{E}}}{\rm{\Delta }}t$ is the membrane potential change caused by the injected currents during the period. The LCM uses equation (4) to calculate the membrane potentials of neuron groups iteratively. The membrane potential change Δ*V* is assumed to be the aggregate postsynaptic potential (PSP) at the soma, and is determined by three processes: neuronal firing, synaptic transmission and postsynaptic potential (PSP) aggregation. Each of the three processes is modelled using a set of equations, which are described in details below.

The LCM uses a set of equations to model neuronal processes. For notational convenience, the following conventions are adopted for the equations: (1) the LCM treats the mean (somatic) membrane potentials of neuron groups (denoted by *V*) as state variables; (2) the LCM uses spike rates (denoted by *ϕ* or *F*) to measure the interaction between neuron groups. The spike rate is defined as the average number of spikes a neuron of one group receives from another group per unit time; (3) neuron groups can be differentiated by neuron type (as listed in Table S1 in the Supplementary Information) or column number. Subscripts *q* or *p* may be used to denote a neuron group in a column. For synaptic transmission, *p* and *q* respectively indicate the presynaptic and postsynaptic neuron group; (4) the LCM simulates PSPs (denoted by *PSP*) generated through effects on different neurotransmitter receptors. The superscript [rcpt] denotes the receptor type; (5) Subscripts and superscripts may be omitted when the equation applies to all relevant objects in the same class (such as neuron groups, receptors, or synaptic connections) equally but parameter values may differ.

The LCM determines the mean spike rate generated by a neuron group using a sigmoid function^16,38^:

5 $$F(t)=\frac{{F}^{[\max ]}}{1+\exp [\,-\,k(V-{V}^{[\mathrm{HMF}]})]},$$

where *F*(*t*) is the mean spike rate, *k* is the firing gain, *F*^[max]^ is the maximum firing rate of the neuron groups, and *V*^[HMF]^ is the membrane potential at half maximum firing rate. Equation (5) is similar in behaviour to a power-law function at low voltage^39^ and converges to *F*^[max]^ at high voltage. The LCM also incorporates time delays of spike propagation. The time delay for a spike to reach a postsynaptic neuron group is calculated with:

6 $${\rm{\Delta }}{t}^{[\mathrm{AP}]}={s}^{[\mathrm{axon}]}/{v}^{[{\rm{AP}}]},$$

where *s*^[axon]^ is the length of the axon connecting the two neuron groups, and *v*^[AP]^ is the spike propagation speed, which is set to 1.0 m/sec^6^. In the LCM, axon length is assumed to be the distance between the two groups (i.e. direct synaptic connections between neuron groups), and spike rates remain constant during propagation.

Afferent spikes change the membrane potentials of the target neuron groups. The amplitude of the postsynaptic potential (PSP) depends on the afferent spike rate and the membrane potential of the target neuron group. PSP amplitude is determined by^16^:

7 $$PS{P}_{qp,0}^{[\mathrm{rcpt}]}({V}_{q},{\varphi }_{p})=-\,{g}_{p}{N}_{qp}({V}_{q}-{V}_{p}^{[\mathrm{rev}]})\,{\varphi }_{p}\,\exp (\,-\,{\lambda }^{[\mathrm{rcpt}]}{\varphi }_{p}),$$

where the subscript 0 denotes the PSP amplitude at the synapse (see below), *ϕ*_*p*_ is the afferent spike rate (see below), *g*_*p*_ is the synaptic gain at the resting membrane potential (referred to as synaptic gain), *N*_*qp*_ is the synapse number between the two neuron groups, ${V}_{p}^{[\text{rev}]}$ is the reversal membrane potential (0 for excitatory spikes and −70 mV for inhibitory spikes) and *λ*^[rcpt]^ is the spike adaptation parameter. Three kinds of neurotransmitter receptors were considered: *AMPA*, *NMDA*, and *GABA* receptors. The excitatory synaptic gain (*g*_E_) controls the PSPs of both AMPA and NMDA receptors, and inhibitory synaptic gain (*g*_I_) controls PSP of the GABA receptors. The time course of the PSP is modelled with modified bi-exponential functions:

8 $${R}^{[{\rm{rcpt}}]}(t)=A\,\exp (-\frac{t-{\tau }_{0}^{[{\rm{rcpt}}]}}{{\tau }_{f}^{[{\rm{rcpt}}]}})[1-\exp (-\frac{t-{\tau }_{0}^{[{\rm{rcpt}}]}}{{\tau }_{r}^{[{\rm{rcpt}}]}})]H(t-{\tau }_{0}^{[{\rm{rcpt}}]}),$$

where ${\tau }_{0}^{[{\rm{rcpt}}]}$ is the synaptic transmission delay, ${\tau }_{r}^{[{\rm{rcpt}}]}$ and ${\tau }_{f}^{[{\rm{rcpt}}]}$ are the time constants respectively controlling the rise and fall of the PSP, $A=({\tau }_{f}^{[{\rm{rcpt}}]}+{\tau }_{r}^{[{\rm{rcpt}}]})/{({\tau }_{f}^{[{\rm{rcpt}}]})}^{2}$ is the normalising constant, and *H*(*x*) is a Heaviside step function, with a value of 1 when *x* ≥ 0 and 0 elsewhere. The LCM also considers the time delay for PSPs to reach the neuron body using an approach similar to Equation (6). It also assumes that PSP amplitudes decay exponentially during propagation, i.e.:

9 $$PS{P}_{qp}=PS{P}_{qp,0}\,\exp (\,-\,{s}^{[\mathrm{dend}]}/{\lambda }^{[{\rm{PSP}}]}),$$

where *PSP*_*qp*,0_ is the PSP amplitude at the synapse, *PSP*_*qp*_ is the corresponding membrane change in neuron body, *s*^[dend]^ is the distance between the synapse and the neuron body, and *λ*^[PSP]^ is the PSP decay factor. The change in membrane potential in the target neuron group is the aggregate of all PSPs^16^:

10 $${\rm{\Delta }}{V}_{q}=\sum _{p}\,\sum _{[{\rm{rcpt}}]}\,PS{P}_{qp}^{[\mathrm{rcpt}]}({V}_{q},{\varphi }_{p})\otimes {R}^{[\mathrm{rcpt}]}(t),$$

where ⊗ denotes convolution in time, and aggregation is performed over all receptors and presynaptic neuron groups in all columns.

The model incorporates the laminar architecture of the cortex represented as five laminae (layers I to VI with layers II and III combined). The cortex is divided horizontally into columns containing eleven neuron groups spanning the five cortical layers (see Fig. 1). The model uses a quantitative synaptic connection map to define the synaptic connections between neuron groups (see Table S5 in Supplementary Information). This map was derived from the literature^40^. Each entry in the map represents the average number of synapses on a neuron that project from a specific type of neuron. A neuron may receive thousands of synaptic connections from many surrounding cortical columns. The synaptic connection map only shows the types of presynaptic neurons, and does not contain information on columnar origin. The LCM also assumes that the density of presynaptic neurons connected to a specific neuron is normally distributed according to the following equation:

11 $$r({s}_{qp})=\frac{X}{2\pi {({\sigma }^{[\mathrm{synp}]})}^{2}}\exp (-\frac{{s}_{qp}^{2}}{2{({\sigma }^{[\mathrm{synp}]})}^{2}}),$$

where *r*(*s*_*qp*_) is the proportion of synapses from neurons located at a distance *s*_*qp*_ from the target neuron; *σ*^[synp]^ is the standard deviation of the spatial distribution of the presynaptic neurons, set to 80 um for pyramidal neurons^41^ and 40 um for spiny stellate and inhibitory neurons. *X* is a Gaussian random number with mean of 1 and standard deviation of 0.2, to avoid unrealistic synaptic connections that are totally symmetric.

The LCM also incorporates the thalamocortical loop. The thalamus is modelled as an additional layer to the previously described cortical model, allowing the propagation delay between the thalamus and cortex to be simulated. Three groups of thalamic neurons are considered: interneurons in the reticular nucleus of the thalamus (IRTN), relay neurons in the lateral geniculate nucleus (RLGN), and interneurons in the lateral geniculate nucleus (ILGN). Synaptic connections formed between thalamic neuron groups and cortical neuron groups are added. The source code of the LCM may be downloaded from the GitHub website (http://github.com/jiaxin-du).

### Simulation parameters

A large number of parameters is used to describe the behaviour of neuron groups and their connections. These are divided into four categories: (1) parameters that describe the physiological properties of neuron groups (see Table S3 in the Supplementary Information); (2) parameters that describe the properties of synapses and receptors (see Table S4 in the Supplementary Information); (3) parameters that describe cortical structure (see Table S2 in the Supplementary Information), and (4) parameters that describe synaptic connections between neuron groups (see Table S5 in the Supplementary Information). Most parameter values are based on published experimental data. We note that the physiological parameters are not independent and that changes in one parameter can usually be compensated by changes in other parameters (refer to our previous paper^14^).

### Data analysis

To determine the excitation level of a cortical column, we calculate the mean firing rates of excitatory neuron groups in the column, from which the MFRs, PSDs and MSCs of neuronal firing are determined. The MFRs and PSDs were estimated from the firing rates in the central column, and the MSCs were estimated from these in the central 100 columns. The MFRs were the temporal mean of firing rates weighted by a tapered cosine window function, which was used to minimise the cut-off effects of oscillatory firing rates. The PSDs and MSCs of firing rates were estimated using Welch’s method^42^. The firing rates were first split into ten segments of equal length with 50% overlap between consecutive segments. Each segment was then multiplied by a Hamming windowing function. PSDs and MSCs were determined from the windowed segments using a fast Fourier transform algorithm as implemented in the FFTW package^43^. The MSCs of firing rates in columns were estimated using

12 $$\mathrm{MSC}(f)=\frac{2}{N(N-1)}\,\sum _{i=1}^{N}\,\sum _{j=i+1}^{N}\,\frac{|{\rm{CPSD}}({F}_{i},{F}_{j}){|}^{2}}{{\rm{PSD}}({F}_{i})\cdot {\rm{PSD}}({F}_{i})},$$

where PSD(*F*_*i*_) computes the PSDs of firing rates in column *i* (*F*_*i*_); CPSD(*F*_*i*_, *F*_*j*_) computes the cross power spectrum densities between *F*_*i*_ and *F*_*j*_; and *N* = 100 is the number of columns used for MSC calculation. The final PSDs and MSCs were the mean PSDs and MSCs of the segments, respectively.

## Supplementary information

Supplementary Information
