# Supplementary material for: Small changes in synaptic gain lead to seizure-like activity in neuronal network at criticality
Source: Sci Rep. 2019 Jan 31;9:1097. doi: 10.1038/s41598-018-37646-9 (PMC6355815; doi:10.1038/s41598-018-37646-9)
Supplement: Supplementary file 1 — Supplementary Information [file 41598_2018_37646_MOESM1_ESM.doc]

# Small changes in synaptic gain lead to seizure-like activity in neuronal network at criticality.

Jiaxin Du*, Viktor Vegh, and David C Reutens

The University of Queensland, Centre for Advanced Imaging, St Lucia QLD 4072 Australia

*To whom correspondence may be addressed. E-mail: jiaxin.du@cai.uq.edu.au or jiaxin.du@outlook.com

# Supplementary Tables

Table S1 Neuron groups modelled in the LCM.

| Acronym | Neuron group | % of cortical neurons^†^ |
| --- | --- | --- |
| **E1** | Excitatory neuron in cortical layer I | 0.05 |
| **I1** | Inhibitory neuron in cortical layer I | 1.5 |
| **P2/3** | Pyramidal neuron in cortical layer II/III | 26.3 |
| **I2/3** | Inhibitory neuron in cortical layer II/III | 7.4 |
| **P4** | Pyramidal neuron in cortical layer IV | 9.3 |
| **SS4** | Spiny stellate neuron in cortical layer IV | 18.6 |
| **I4** | Inhibitory neuron in cortical layer IV | 7.0 |
| **P5** | Pyramidal neuron in cortical layer V | 6.2 |
| **I5** | Inhibitory neuron in cortical layer V | 1.4 |
| **P6** | Pyramidal neuron in cortical layer VI | 18.3 |
| **I6** | Inhibitory neuron in cortical layer VI | 4.0 |
| **IRTN** | Interneurons in RTN of the thalamus |  |
| **RLGN** | Relay neurons in LGN of the thalamus |  |
| **ILGN** | Interneurons in LGN of the thalamus |  |

^†^ The data are derived from the paper[^1^](#_ENREF_1). Acronyms: **LGN**–lateral geniculate nucleus; **RTN**–reticular nucleus.

Table S2 Parameters for thalamo-cortical structures used in the LCM ^†^.

|  | Layer | Depth (mm) |
| --- | --- | --- |
| **Cortex** | I | 0-0.166 |
|  | II & III | 0.166-0.631 |
|  | IV | 0.631-1.141 |
|  | V | 1.141-1.278 |
|  | VI | 1.278-1.622 |
| **Thalamus** | TH | 20-22 |

^†^ The data are derived from the paper[^1^](#_ENREF_1).

Table S3 Parameters for electrophysiological properties of neurons^†^.

| Symbol | Meanings | Values |
| --- | --- | --- |
| $V_{q}^{[0]}$ | Resting membrane potential of neuron groups | -65 mV [^2^](#_ENREF_2) |
| $V_{q}^{[\text{rev}]}$ | Reverse membrane potential of neuron groups | Excitatory groups: 0 mV  Inhibitory groups: -70 mV |
| $V_{q}^{[\text{VHM}]}$ | Voltage at half maximum firing rates (VHM) | -50 mV (^‡^) |
| $k_{q}$ | Firing gain for neuron groups | 0.33 mV^-1^ |
| $v_{q}^{[AP]}$ | Propagation speed of spikes along axons | 1.0 m/s [see, for example^, 3^](#_ENREF_3) |
| $v_{p}^{[PSP]}$ | Propagation speed of postsynaptic potentials (PSP) along dendrites | 0.2 m/s [adapted from Fig. 2 i^n 4^](#_ENREF_4) |
| $\lambda_{q}^{[\text{PSP]}}$ | PSP decay constant | 1.6 mm [adapted from Fig 1 i^n 4^](#_ENREF_4) |
| $\sigma^{\text{[synp]}}$ | Standard deviation (SD) of the distance from pre-synaptic neuron to post-synaptic neuron | Excitatory synapse: 80 um [^5^](#_ENREF_5)  Inhibitory synapse: 40 um |

^†^ The references from which the parameter values were derived are cited above. If no reference is given, the value was set empirically or inherited from the continuum cortex model[^6^](#_ENREF_6).

^‡^ Values for firing gain and VHM were chosen to fit the power law firing function $Q_{q}=k(\mathrm{V}-V_{q}^{[0]})^{n}$ at low membrane potential ($V_{q}<-50$mV), where $k=0.015$ and $n=3$. The parameter values for the power law are derived from the work of Hansel and Vreeswijk[^7^](#_ENREF_7).

Table S4 Parameters for synaptic transmission.

| Symbol | Meanings | Values |
| --- | --- | --- |
| $g_{p}^{[\mathrm{rcpt}]}$ | Synaptic transmission gain | free parameter, see main text |
| $\lambda_{p}^{[\text{rcpt}]}$ | Spike adaption factor of receptor | AMPA: 0.012 [^6^](#_ENREF_6)  NMDA: 0.037 [^6^](#_ENREF_6)  GABA: 0.005 [^6^](#_ENREF_6) |
| $\tau^{\text{[rcpt]}}$ | Synaptic transmission delay | Excitatory groups: 0.38 msec [^8^](#_ENREF_8)  Inhibitory groups: 0.9 msec [^8^](#_ENREF_8) |
| $\tau_{r}$ | PSP rise time | AMPA: 2.6 msec^†^ [^9^](#_ENREF_9)  NMDA: 6.6 msec^†^ [^10^](#_ENREF_10)  GBBA: 3 msec^†^ [^11^](#_ENREF_11) |
| $\tau_{f}$ | PSP fall time | AMPA: 13 msec^†^  NMDA: 60 msec^†^  GABA: 12.5 msec^†^ |

^†^ The PSP rise and fall times are derived from experimentally measured 10% to 90% rise time and half width of the PSP time course.

Table S5 Numbers of synapses between neuron types ^†^.

| post-synaptic | loc^1^ | Presynaptic neurons | | | | | | | | | | | | | | | |
| --- | --- | --- | --- | --- | --- | --- | --- | --- | --- | --- | --- | --- | --- | --- | --- | --- | --- |
|  |  | **E1** | **I1** | **P2/3** | **I2/3** | **P4** | **SS4** | **I4** | **P5** | **I5** | **P6** | **I6** | **PLGN** | **ILGN** | **IRTN** | **CC**^2^ | **SI**^3^ |
| **E1** | L1 | 907 | 1600 | 907 | 160 |  |  |  |  |  |  |  | 408 |  |  | 7752 |  |
| **I1** | L1 | 73 | 898 | 560 | 151 | 9 |  |  | 9 |  |  |  | 364 |  |  | 6689 |  |
| **P2/3** | L1 |  | 133 | 82 | 16 | 1 |  |  | 1 |  |  |  | 54 |  |  | 1019 |  |
|  | L2/3 |  |  | 3474 | 783 | 447 | 435 | 46 | 429 |  | 133 | 46 |  |  |  |  |  |
| **I2/3** | L2/3 |  | 54 | 1769 | 509 | 226 | 217 | 28 | 215 |  | 69 | 23 | 22 |  |  | 408 |  |
| **P4** | L1 |  | 82 | 51 | 10 | 1 |  |  | 1 |  |  |  | 33 |  |  | 629 |  |
|  | L2/3 |  |  | 546 | 80 | 70 | 68 | 5 | 68 |  | 22 | 7 |  |  |  |  |  |
|  | L4 |  |  | 216 | 40 | 211 | 760 | 468 | 65 |  | 1585 | 297 | 151 |  |  | 1233 |  |
| **SS4** | L4 |  |  | 218 | 53 | 226 | 828 | 496 | 56 |  | 1723 | 305 | 162 |  |  | 1329 |  |
| **I4** | L4 |  |  | 168 | 39 | 138 | 497 |  | 35 |  | 1024 | 182 | 95 |  |  | 789 |  |
| **P5** | L1 |  | 138 | 85 | 16 | 1 |  |  | 1 |  |  |  | 55 |  |  | 1054 |  |
|  | L2/3 |  |  | 388 | 57 | 50 | 48 | 4 | 48 |  | 15 | 5 |  |  |  |  |  |
|  | L4 |  |  | 12 | 3 | 18 | 68 | 28 | 4 |  | 143 | 25 | 14 |  |  | 110 |  |
|  | L5 |  |  | 2040 | 92 | 334 | 237 | 39 | 567 | 85 | 202 | 517 | 25 |  |  | 345 |  |
| **I5** | L5 |  |  | 1356 | 75 | 224 | 158 | 33 | 376 | 66 | 128 | 340 | 15 |  |  | 215 |  |
| **P6** | L1 |  | 6 | 4 | 1 |  |  |  |  |  |  |  | 3 |  |  | 48 |  |
|  | L2/3 |  |  | 102 | 15 | 13 | 13 | 1 | 13 |  | 4 | 1 |  |  |  |  |  |
|  | L4 |  |  | 42 | 12 | 63 | 240 | 100 | 13 |  | 505 | 88 | 48 |  |  | 390 |  |
|  | L5 |  |  | 405 | 10 | 67 | 48 | 5 | 112 | 12 | 38 | 101 | 4 |  |  | 64 |  |
|  | L6 |  |  | 96 | 8 | 50 | 61 | 4 | 192 | 12 | 552 | 593 | 134 |  |  | 2137 |  |
| **I6** | L5 |  |  | 1356 | 75 | 224 | 158 | 33 | 376 | 66 | 128 | 340 | 15 |  |  | 215 |  |
|  | L6 |  |  | 81 | 6 | 42 | 52 | 3 | 161 | 13 | 464 | 496 | 113 |  |  | 1794 |  |
| **IRTN** | TN |  |  |  |  |  |  |  |  |  | 1200 |  | 800 |  | 400 |  |  |
| **PLGN** | TN |  |  |  |  |  |  |  | 712 |  | 884 |  | 284 | 200 | 1036 |  | 284 |
| **ILGN** | TN |  |  |  |  |  |  |  | 222 |  | 276 |  | 15 | 732 |  |  | 1461 |

^†^ Each number in the table represents the number of synapses on a neuron projecting from neurons of the indicated type. Data derived from . For completeness, all synaptic connections identified by [Binzegger, et al^. 12^](#_ENREF_12) are listed. The LCM only incorporated synapse numbers no smaller than 10, and the shadowed connections in the table were omitted. ^1^ Synapse locations: **L1-L6**, cortical layer I to VI; **TN**, thalamus. ^2^ **CC** stands for cortico-cortical connections; ^3^ **SI** stands for sensory inputs to the thalamus.

# References

1 Beaulieu, C. & Colonnier, M. The number of neurons in the different laminae of the binocular and monocular regions of area 17 in the cat, Canada. *J. Comp. Neurol.* **217**, 337-344, doi:10.1002/cne.902170308 (1983).

2 Carandini, M. & Ferster, D. Membrane potential and firing rate in cat primary visual cortex. *J. Neurosci.* **20**, 470-484 (2000).

3 Swadlow, H. A. Efferent neurons and suspected interneurons in motor cortex of the awake rabbit: axonal properties, sensory receptive fields, and subthreshold synaptic inputs. *J. Neurophysiol.* **71**, 437-453 (1994).

4 Stuart, G. J. & Sakmann, B. Active propagation of somatic action potentials into neocortical pyramidal cell dendrites. *Nature* **367**, 69-72, doi:10.1038/367069a0 (1994).

5 Song, S., Sjostrom, P. J., Reigl, M., Nelson, S. & Chklovskii, D. B. Highly nonrandom features of synaptic connectivity in local cortical circuits. *PLoS Biol.* **3**, e68, doi:10.1371/journal.pbio.0030068 (2005).

6 Wright, J. J. Generation and control of cortical gamma: findings from simulation at two scales. *Neural Netw* **22**, 373-384, doi:10.1016/j.neunet.2008.11.001 (2009).

7 Hansel, D. & van Vreeswijk, C. How noise contributes to contrast invariance of orientation tuning in cat visual cortex. *J. Neurosci.* **22**, 5118-5128 (2002).

8 Katz, B. & Miledi, R. The Measurement of Synaptic Delay, and the Time Course of Acetylcholine Release at the Neuromuscular Junction. *Proc R Soc Lond B Biol Sci* **161**, 483-495 (1965).

9 Thomson, A. M., Deuchars, J. & West, D. C. Large, deep layer pyramid-pyramid single axon EPSPs in slices of rat motor cortex display paired pulse and frequency-dependent depression, mediated presynaptically and self-facilitation, mediated postsynaptically. *J. Neurophysiol.* **70**, 2354-2369 (1993).

10 Hestrin, S., Nicoll, R. A., Perkel, D. J. & Sah, P. Analysis of Excitatory Synaptic Action in Pyramidal Cells Using Whole-Cell Recording from Rat Hippocampal Slices. *J Physiol-London* **422**, 203-225 (1990).

11 Thomson, A. M. & Deuchars, J. Synaptic interactions in neocortical local circuits: dual intracellular recordings in vitro. *Cereb. Cortex* **7**, 510-522, doi:DOI 10.1093/cercor/7.6.510 (1997).

12 Binzegger, T., Douglas, R. J. & Martin, K. A. A quantitative map of the circuit of cat primary visual cortex. *J. Neurosci.* **24**, 8441-8453, doi:10.1523/JNEUROSCI.1400-04.2004 (2004).

13 Izhikevich, E. M. & Edelman, G. M. Large-scale model of mammalian thalamocortical systems. *Proc Natl Acad Sci U S A* **105**, 3593-3598, doi:10.1073/pnas.0712231105 (2008).
